# Competition between Cognitive Control and Encapsulated, Unconscious Inferences: Are Aha-Experiences Special?

**DOI:** 10.3389/fpsyg.2016.00626

**Published:** 2016-05-02

**Authors:** Donish Cushing, Anthony G. Velasquez, Ezequiel Morsella

**Affiliations:** ^1^Department of Psychology, San Francisco State UniversitySan Francisco, CA, USA; ^2^Department of Neurology, University of California, San FranciscoSan Francisco, CA, USA

**Keywords:** mind wandering, mindfulness, consciousness, unconscious inference, aha experience

What are the conditions under which a mental state such as mind wandering can help problem solving? By addressing this question, Zedelius and Schooler (2015) contribute to our understanding of the dynamics between distinct modes of thinking (i.e., mindfulness vs. mind wandering) and different kinds of problem solving (i.e., analytic strategy vs. insight learning). To place this important contribution within a wider context, it is important to appreciate that the contents composing the “conscious field,” defined here as all that one is aware of at one moment in time, are, in most cases, generated by sophisticated processes transpiring beneath the horizon of consciousness (Morsella et al., in press). For example, to the self, the color of objects, depth perception, urges, and dream content “just happen.” Accordingly, Helmholtz (1856/1925) speaks of such *conscious contents* as arising from “unconscious inferences,” which, for him, include even high-level contents, such as the phonological forms activated by automatic word reading.

If unconscious inferences engender the vast majority of conscious contents, including low-level contents (e.g., nausea) and high-level contents (e.g., automatic word reading and “earworms”), then why is the Aha-experience so special and, to the self, so startling? It seems that the Aha-experience is special for reasons other than its being involuntary and sophisticated, which are properties shared by other unconscious inferences. The findings reported by Zedelius and Schooler (2015) begin to illuminate these properties and also the underlying mechanisms and conditions responsible for them.

That Aha-experiences are engendered in a manner resembling that of most other conscious contents can be appreciated in the following dream scenario, which was experienced by one of us (EM). One is dreaming that one is seated at a desk, trying to title a piece of work, but is distracted by an incessant earworm. After some time, and despite the earworm, a solution springs to mind. In this example, it is evident that, though the Aha-experience is linked to only one conscious content (the thought of the title), that content is not the only content that is generated by unconscious, sophisticated processes: The percept of the desk, the urge to title the work, and the undesired earworm all “just happen” to the self in the dream (Morsella et al., in press). In terms of the intentional nature of their creation, these contents are the same.

From this standpoint, the color of an apple, which is traditionally regarded as “stimulus driven,” and, say, becoming annoyed upon hearing a silly comment, are similar regarding the unintentional nature of the introduction of these contents into the conscious field. Yet, the former is regarded as a property of a stimulus, and the latter is not. Instead, the latter is regarded as being more than that—as *one's response* to a stimulus. This lay intuition suggests that the feeling of annoyance was generated in a manner that is more intentional than that of the perception of the color red. But this is not the case: both contents “just happen” to the self.

Because of insuppressible, unconscious inferences, conscious contents often stem directly from the immediate environment and seldom from long, complicated chains of thought. Accordingly, in several studies (see Godwin et al., 2013), subjects performed a concentration exercise (9 min) requiring them to focus on their breathing. During this task, they also had to observe spontaneous thoughts and count the number of cognitions/percepts (“links”) that they believed led to each spontaneous thought. On average, subjects reported less than two links per thought, and over 80% of the thoughts were attributed to a known cause. Important for present purposes, roughly half of the thoughts were attributed to stimuli in the present environment.

To place the contribution by Zedelius and Schooler (2015) within a wider context, it is also important to appreciate that, even when problem solving does not involve an Aha-experience, and stems from intentional, directed thought (e.g., analytic strategy), the problem solving still stems in part from sophisticated, unconscious processes, which are never mediated consciously. A good analogy involves the control of action. One is never conscious of the sophisticated patterns of efference to the muscles (Jeannerod, 2006), even when first executing an action, when the action is believed to be the most “consciously mediated.” (Well-learned, repeated actions are regarded as being less consciously-mediated.) For example, for both the novice piano player and the expert pianist, the motor efference responsible for the playing of each note is always consciously-impenetrable. Similarly, each content composing a stream of consciousness, whether the stream is mind wandering or directed thought, stems in large part from processes that are consciously-impenetrable (e.g., lexical retrieval and syntax; Merrick et al., 2015).

Of course, one can influence content generation through sophisticated techniques such as *indirect cognitive control* (Morsella et al., 2009), such as when one imagines something scary in order to induce fear, whose generation is usually encapsulated. Similarly, one can impede the generation of a particular conscious content (e.g., an earworm) by intentionally occupying its content generator with some other activity (e.g., reciting a poem). (Research reveals that such a strategy is limited; Cho et al., 2014.) This blocking effect might contribute to the finding by Zedelius and Schooler (2015) that high levels of mindfulness (toward some content) interfere with the generation of out-of-the-blue contents, such as those associated with Aha-experiences. Nevertheless, techniques such as indirect cognitive control occur usually only in adult humans and are the exception rather than the rule regarding the nature of content generation. Most conscious contents “just happen.” For reasons yet to be specified, they are not accompanied by any Aha-experiences.

To answer the question posed in our title, Aha-experiences are special, but for reasons other than the unintentional and sophisticated nature of their generation. The groundbreaking research by Zedelius and Schooler (2015) begins to illuminate what is special about these peculiar conscious contents and how their generation depends upon certain factors (e.g., low mindfulness and high mind wandering).

## Author contributions

Each author contributed to the writing of this manuscript and to the theorizing presented in it. DC and AV wrote the first draft of the piece.

### Conflict of interest statement

The authors declare that the research was conducted in the absence of any commercial or financial relationships that could be construed as a potential conflict of interest. The reviewer LC and handling Editor declared their shared affiliation, and the handling Editor states that the process nevertheless met the standards of a fair and objective review.
